# Treatment of stable slipped capital femoral epiphysis: systematic review and exploratory patient level analysis

**DOI:** 10.1007/s10195-017-0469-4

**Published:** 2017-08-22

**Authors:** H. Naseem, S. Chatterji, K. Tsang, M. Hakimi, A. Chytas, S. Alshryda

**Affiliations:** 1 Royal Manchester Children’s Hospital, Central Manchester Hospitals Foundation Trust, Oxford Road, Manchester, UK; 2 University Hospitals of North Midlands, Royal Stoke Hospital, Stoke-on-Trent, UK

**Keywords:** Slipped upper femoral epiphysis, Stable, SUFE, SCFE, Unstable, Pinning in situ, Ganz surgical dislocation

## Abstract

**Background:**

Several aspects of slipped capital femoral epiphysis (SCFE) treatment remain controversial. Loder’s work has been instrumental in changing our understanding and approach to the management of the condition when he introduced the concept of “slip instability” and showed that avascular necrosis (AVN) developed in 47% of unstable slips but none of the stable slips. As the two types of SCFE behave differently in terms of presentation, progress and complications, we approached them as two different conditions to highlight these differences. This paper focuses on treatments of stable SCFE.

**Materials and methods:**

An extensive literature search was carried out from multiple databases. One thousand six hundred and twenty-three citations were screened. Three hundred and sixteen full publications were obtained for further scrutiny. Fifty-eight studies (2262 hips) were included in the review. These studies evaluated 6 interventions. AVN was chosen as a surrogate for bad outcome. Secondary outcomes were chondrolysis (CL), femoro-acetabular impingement (FAI), osteoarthritis (OA) and patients’ reported outcomes. The latter were pooled when they met our predefined criteria.

**Results:**

The type of surgical intervention was an important risk factor. Pinning in situ (PIS) was associated with the lowest AVN rate (1.4%). Moreover, the CL, FAI and OA rates were relatively low in patients who underwent PIS. These were not translated into high patient satisfaction rates among these patients, with only 47% reporting an “excellent” outcome. In contrast, 87% of patients who underwent Ganz surgical dislocation reported an “excellent” outcome. The Ganz surgical dislocation was associated with an AVN rate of 3.3%; double that observed in pinning in situ.

**Conclusion:**

Pinning in situ is the best treatment for mild and moderate stable slip. Ganz surgical dislocation gives higher patient satisfaction for severe stable slip but the risk of AVN is doubled compared with pinning in situ. Devices that allow continued growth may be better than standard screws.

**Level of evidence:**

Level III.

## Introduction

Slipped capital femoral epiphysis (SCFE) is an uncommon paediatric hip disorder occurring at an incidence of 1–10/100,000. Despite it being uncommon, it is a condition which is important not to miss, as suboptimal management can lead to substantial disability. Various theories regarding the pathophysiology of this condition have been proposed and include increased shear forces acting on a weakened physis. Mechanical and hormonal factors have both been implicated [1].

Loder’s work has been instrumental in changing our understanding and approach to the management of the condition. In a landmark paper [2], he categorised SCFEs into stable and unstable based on the patient’s ability to ambulate (with or without crutches) or not. Almost half the patients with an unstable slip developed poor outcomes versus none in the stable group. This finding has been confirmed by several authors [3–6]. Although our knowledge of the condition has advanced over the last three decades, this has not translated into obviously better outcomes [7–20].

Various treatment options have been proposed, including bone peg epiphysiodesis, pinning in situ (PIS), closed reduction and pinning (CRIF), open reduction and physeal osteotomy (PO), open reduction and internal fixation (ORIF) and Ganz surgical dislocation (GSD). The types of fixation devices and their designs have been the subject of various researches. Ideal fixation devices should prevent further slippage, while allowing for continued growth with possible remodelling and prevention of future impingement [21–24]. The general consensus appears to be managing patients according to their slip grade. This was addressed in a review of the subject by Loder et al. in 2012, with mild and moderate slips tending to be treated with pinning in situ. Severe slips can be challenging to manage, as achieving a screw position centrally in the epiphysis with PIS may be technically difficult and subsequent remodelling may be insufficient.

With a low incidence, several treatment options and a potential lack of appropriate outcome measures, performing adequately powered randomised controlled trials (RCTs) is challenging. A nationwide study is currently underway, supported by the British Society of Children’s Orthopaedic Surgery (the BOSS study), to help pave the way for future large-scale RCTs to inform decision making [25].

Given the substantial differences in the outcomes between stable and unstable slips we have chosen to study them separately, conducting two systematic reviews and patient level analysis. In a previous study we dealt with the outcomes of various interventions in treating unstable slips [26] and in this study we have critically appraised the published research to provide evidence on what may be the best current treatment for a stable slipped capital femoral epiphysis.

## Materials and methods

This is a systematic review and patient level analysis of studies assessing the outcomes of interventions in stable slipped capital femoral epiphysis. As the concept of slip stability was introduced in 1993, studies before this date were not included. The work was conducted as part of a Cochrane Review and followed a prospective review protocol [27]. Reporting follows the PRISMA guidelines [28].

Avascular necrosis of the femoral head (AVN) was chosen as a surrogate for a poor outcome; this was our primary outcome measure [1, 29]. The secondary outcome measures selected were osteoarthritis (OA), chondrolysis (CL), femoro-acetabular impingement (FAI) and surgical complications such as metalware problems, nerve palsy and infection. Several studies used patient reported outcome measures (PROMs) and these were also included in the analysis.

A hierarchical approach was used to include relevant studies. Randomised controlled trials (RCTs) or controlled clinical trials (CCTs) were included if adequately informative, otherwise inclusion would be firstly extended to controlled observational designs and secondly to other uncontrolled designs such as case series.

An extended literature search was performed of the following databases: Cochrane Bone, Joint and Muscle Trauma Review Group Specialised Register, the Cochrane Central Register of Controlled Trials (The Cochrane Library, current issue), MEDLINE (1993–2016), EMBASE (1993–2016), CINAHL (1993–2016), and Science Citation Index (ISI Web of Science 1993–2016). Table 1 summarises the search strategy for MEDLINE, which was modified for the other databases. The bibliographies of the retrieved literature were cross-referenced to identify other relevant studies.

**Table 1:** Search strategies

1. Epiphyses, Slipped/
2. (slipped adj3 upper adj3 femoral adj3 epiphysis).tw.
3. Femur Head/ab, pa, su [Abnormalities, Pathology, Surgery]
4. exp Femur Neck/ab, pa, su [Abnormalities, Pathology, Surgery]
5. SUFE.tw.
6. (slipped adj3 epiphyses).tw.
7. exp Slipped Capital Femoral Epiphyses/
8. SCFE.mp. or SCUFE.tw. [mp = title, abstract, original title, name of substance word, subject heading word, protocol supplementary concept, rare disease supplementary concept, unique identifier]
9. or/1-8
10. randomized controlled trial.pt.
11. controlled clinical trial.pt.
12. randomized.ab.
13. placebo.ab.
14. drug therapy.fs.
15. randomly.ab.
16. trial.ab.
17. groups.ab.
18. or/10-17
19. exp animals/not humans.sh.
20. 18 not 19
21. 9 and 20

The above search strategy was independently applied by two reviewers (HN and SC) to identify studies. The article titles and abstracts were then independently reviewed. Full articles were obtained if the study appeared to be eligible or where this was uncertain. If necessary, authors were contacted for further information and clarification. Our senior authors (KT, AC and SA) were consulted if there was still a disagreement regarding inclusion. If no consensus was reached the study was excluded. Several studies were excluded because they were published more than once with more patients: it was agreed to include the most informative one regardless of the number of patients that were included.

A piloted form was used to extract data independently by two authors (KT and MH). The names of included papers’ authors or institutions were not masked. The data accuracy was jointly double-checked by these two authors and any discrepancies resolved through discussion. The two authors independently assessed the risk of bias in the included studies. The methodological quality of non-randomised studies (NRSs) was assessed using the Newcastle–Ottawa Scale (see Table 2).

**Table 2:** Risk of bias assessment tool for cohort studies

Domain	Items	Maximum number of stars	Notes
Selection	1. Representativeness of the exposed cohort	1	Maximum possible stars is 4
	2. Selection of the non exposed cohort	1	
	3. Ascertainment of exposure	1	
	4. Demonstration that outcome of interest was not present at start of study	1	
Comparability	Comparability of cohorts on the basis of the design or analysis	2	Maximum possible stars is 2
Outcome	1. Assessment of outcome	1	Maximum possible stars is 3
	2. Was follow-up long enough for outcomes to occur	1	
	3. Adequacy of follow-up of cohorts	1	

The continuous data was reported for each trial arm as mean, standard deviation (SD) and group size. We planned to use the mean difference (MD) with corresponding 95% confidence interval (CI) to summarise trial findings and report the treatment effect if the outcomes were measured the same way between trials. The standardised mean difference (SMD) would be used to compare trials that measured the same outcome (construct), but used different scales. The dichotomous data was expressed as proportions or risks, reporting the treatment effect as a risk ratio (RR) with 95% CI. *P* < 0.05 was selected as the level for statistical significance.

Various types of patient satisfaction scores were utilized in the included studies. These are summarised in Table 4. They were categorised into an ordinal scale (excellent, good, fair, poor and failure) by most studies. Authors were not consistent in how they used and reported them. Carlioz et al. [30] used a scale omitting “excellent”. A few authors omitted “failure” in their scales [31–33]. We pooled data as reported in the included studies without assumption or improvisation.

## Results

### Description of studies

**Fig. 1:**
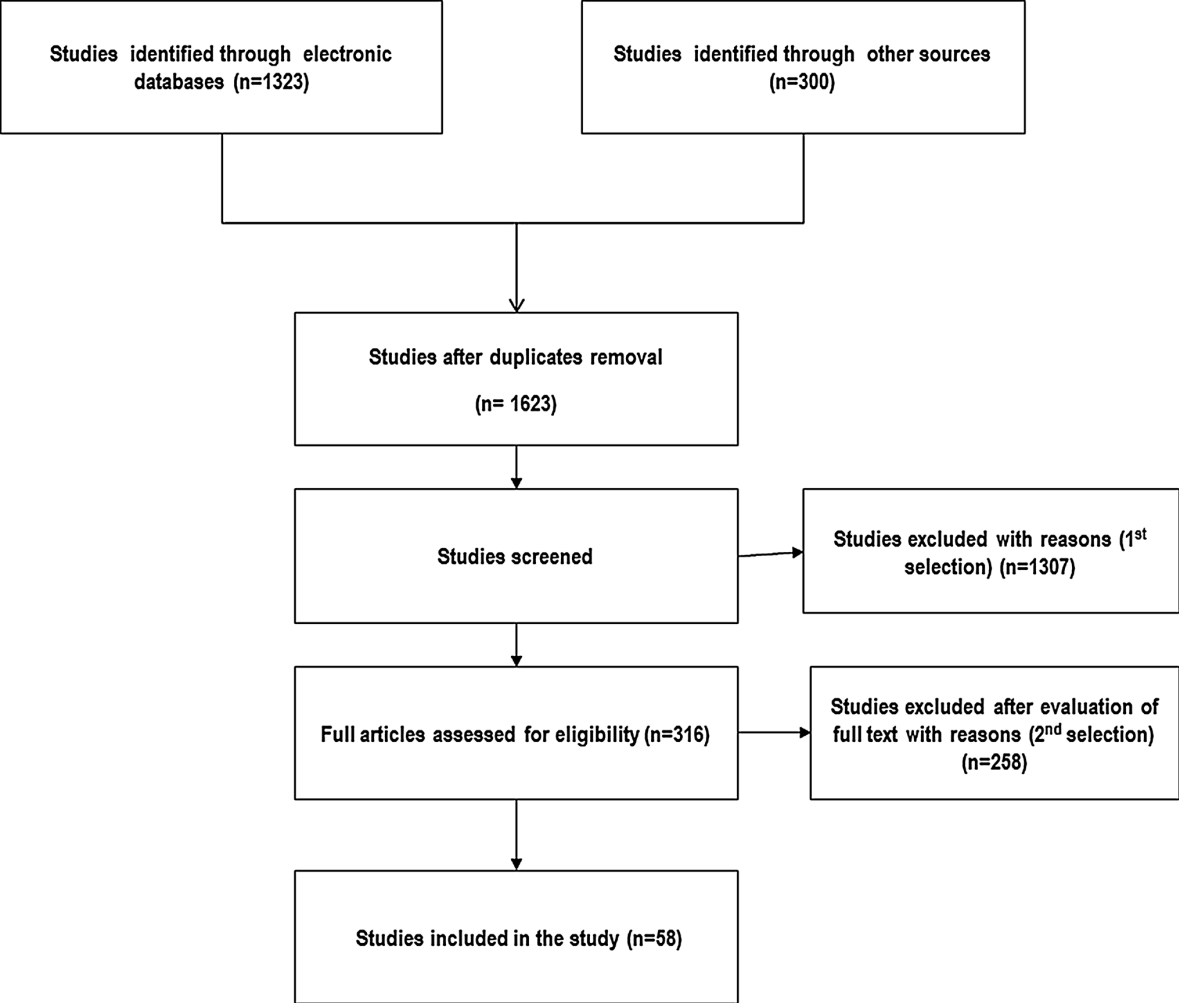
Studies selection flow chart

A total of 1623 potentially relevant citations were identified, of which 1307 were subsequently excluded for reasons such as duplications, reviews and commentaries. The full publications for the remaining 316 citations were obtained and of these 271 studies were further excluded: the main reasons included uncertainty of slip stability, the inability to link patients to outcomes within the study or that the focus of the study was not on outcomes. Forty-five studies were hence used in the review. This process is illustrated in Fig. 1. No RCTs were identified and all were retrospective case series or controlled studies. These scored between 2 and 4 stars (out of 7) on the risk of bias measure.

The treatment options identified were hip spica, bone graft epiphysiodesis, pinning in situ (PIS) pinning using multiple pins, physeal osteotomy (PO) and Ganz surgical dislocation (GSD). Several studies used more than one treatment option. Pinning in situ was the commonest treatment option seen. Patients were excluded from the analysis if there was uncertainty about factors such as the severity of the slip and the occurrence and/or type of reduction. Studies of base of neck osteotomy and intertrochanteric osteotomy were excluded from the review because they included a significant number of healed SCFEs.

In total, 2262 hips drawn from 58 studies were included in the review. Several studies reported on more than one treatment option. Table 3 summarises the included studies and categorises them according to the treatment methods. Three studies reported on 101 hips that were treated with hip spica [34–36] (Table 4). Six studies reported on 464 hips that were treated with bone peg epiphysiodesis [37–42] (Table 5). Nineteen studies (714 hips) reported on pinning in situ using a single screw [3, 23, 30–32, 43–55, 65] (Table 6). Six studies (273 hips) reported on fixation in situ using multiple smooth pins [35, 42, 44, 45, 47, 56] (Table 7). Seventeen studies (615 hips) reported on primary corrective subcapital femoral osteotomy [3, 30, 33, 35, 41, 56–66, 70] (Table 8). Seven studies (95 hips) reported on safe surgical dislocation using the Ganz technique [43, 51, 52, 67–70] (Table 9). Although nine studies reported on screws that allow continued growth, only three met our inclusion criteria [23, 54, 55]. These were further analysed for their effect on growth of the femoral neck.

**Table 3:** Pooled summary of studies of stable slip treatments

Intervention	Hips	AVN (%)	CL (%)	FAI (%)	OA (%)	Satisfactory patients result^a^
Hip spica	101	9.5	20.5	NR	53	NR
Epiphysiodesis	464	3	1.3	NR	23.3	67 (67%) excellent 6 (6%) good 10 (10%) fair 7 (7%) poor 7 (7%) failure
Pinning using single screw	714	1.4	2.1	29.8	3.1	116 (47%) excellent 86 (36%) good 19 (8%) fair 10 (4%) poor 11 (5%) failure
Pinning using multiple pins	273	2.2	4	NR	15	76 (67%) excellent 19 (17%) good 0 (0%) fair 16 (14%) poor 3 (3%) failure
Physeal osteotomy	615	11.1	9.8	1.5	12.2	131 (28%) excellent 210 (45%) good 46 (10%) fair 72 (16%) poor 3 (6%) failure
Ganz surgical dislocation	95	3.1	2.1	6	0	52 (87%) excellent 2 (3%) good 0 (0%) fair 5 (8%) poor 1 (2%) failure

Percentage based on the number of patients in the studies that reported on the relevant outcomes and not the pooled total

*AVN* femoral head osteonecrosis, *CL* chondrolysis, *FAI* femora-acetabular impingement, *OA* osteoarthritis

^a^Satisfactory patients result based on closely related ratings such as Heyman and Herndon classification, Harris hip score or Iowa hip scores, *NR* not reported

**Table 4:** Studies of hip spica treatment in stable slips

Study	Patients	Hips	AVN	CL	FAI	OA	Patient satisfaction^a^	Other complications	Notes
Betz [34]	32	37	0	5	NR	NR	NR		0 acute, 8 acute on chronic and 29 chronic 25 mild, 7 moderate and 5 severe All stable slips
Carney [35]	NR	47	8	6	NR	NR	Mean IHS 65 when SCFEs were reduced and 83 when SCFEs were not reduced		Spica with closed reduction (16 hips) resulted in a mean IHS of 65 points, 6 AVN and 2 CL Spica cast without reduction [26] resulted in a mean IHS of 83 points, 2 AVN and 4 CL
Meier [36]	13	17	NR	10	NR	9	NR	3 pressure sores 3 further slipping	
Total	NR	101	8	21	NR	9	NR		AVN rate 9.5% (8/84). CL rate 20.2% (21/101), FAI (NR), OA 53% (9/17)

*AVN* femoral head osteonecrosis, *CL* chondrolysis, *NR* not reported or suboptimum reporting to provide useful information, *IHS* Iowa hip-rating system; excellent 90–100 points; good 80–89 points; fair 70–79 points; and poor <70 points, *FAI* femora-acetabular impingement, *OA* osteoarthritis, *SCFEs* slipped capital femoral epiphyses

^a^ Satisfactory patients result based on closely related ratings such as Heyman and Herndon classification, Harris hip score or Iowa hip scores

**Table 5:** Studies of using bone peg epiphysiodesis in stable slips

Study	Patients	Hips	AVN	CL	FAI	OA	Patient satisfaction^a^	Other complications	Notes
Adamczyk [38]	225	278	4	0	NR	NR	NR	17 further slipping 4 deep infection 12 re-operation	45 acute, 0 acute on chronic and 278 chronic Outcomes of acute slips were excluded The average length of surgery was 90 min and blood loss was 200 ml. No blood transfusion Iliac crest bone autograft was used
Murray [39]	31	42	4	0	NR	NR	NR	2 re-operation 2 wound healing problems	3 unstable slips were excluded Average operative time was 87 min and blood loss was 148 ml. No blood transfusion Fibular allograft with demineralised bone matrix was used
Rao [37]	43	46	3	2	NR	NR	NR	3 infections 7 cases of transient anterolateral thigh hypesthesia 44 heterotopic ossification	18 unstable (excluded) and 46 stable slips The average operating time and blood loss per hip were 122 ± 34 min and 426 ± 238 ml, respectively
Schmidt [40]	33	40	1	1	NR	NR	35 excellent 1 good 2 fair 2 poor	1 femoral neck fracture 1 sub-trochanteric hip fracture 2 coxa vara	31 mild, 9 moderate, 0 severe 6 unstable and 34 were stable The average time 1 h 57 min and blood loss averaged 360 ml Allograft used
Szypryt [41]	25	30	2	3	NR	7	12 excellent 5 good 8 fair 4 poor	3 wound infection	1 acute, 13 acute-on-chronic, 16 chronic 0 mild, 12 moderate, 18 severe
Zahrawi [42]		28	0	0	NR	NR	20 excellent 0 good 0 fair 1 poor 7 failure	4 wound infection 2 graft failure 1 further slipping 6 needed further surgery	Severity (mean slip angle 30) LOS 21 Duration of surgery 150 min Blood loss 500 ml
Total	NR	464	14	6	NR	NR	67 (68%) excellent 6 (6%) good 10 (10%) fair 7 (7%) poor 7 (7%) failure	16 wound infection 20 further slipping 22 re-operation	AVN rate 3% (14/464). CL rate 1.3% (6/464). FAI (NR), OA 23.3% (7/30)

*AVN* femoral head osteonecrosis, *CL* chondrolysis, *CRIF* closed reduction and internal fixation, *NR* not reported or suboptimum reporting to provide useful information, *LOS* length of stay, *FAI* femora-acetabular impingement, *OA* osteoarthritis

^a^ Satisfactory patients result based on closely related ratings such as Heyman and Herndon classification, Harris hip score or Iowa hip scores

**Table 6:** Studies of pinning in situ using screws

Study	Patients	Hips	AVN	CL	FAI	OA	Patient satisfaction^a^	Other complications	Notes
Abu Amara [65]	NR	37	1	1	30	NR	NR		See physeal osteotomy FAI diagnosis is based on radiological signs. WOMAC (10) HHS (86)
Alshryda [3]	36	36	1	1	NR	NR	NR	1 loss of fixation	Unstable and uncertain hips were excluded. See physeal osteotomy below
Aronson [44]	34	43	1	0	NR	1	27 excellent 12 good 2 fair 2 poor	2 loss of fixation 1 sub-trochanteric fracture 2 failed screw removals	6 acute and 37 chronic 27 mild, 8 moderate and 8 severe See pinning using multiple fine wires
Blanco [45]	80	43	0	0	NR	NR	NR	2 metalware problems 1 reoperation	1 acute, 6 acute on chronic, 36 chronic 23 mild, 12 moderate, 8 severe 1 CRIF See pinning using multiple fine wires
Carlioz [30]	34	38	0	2	NR	NR	31 good 10 fair 2 bad 3 failure	1 sub-trochanteric fracture	6 patients underwent reduction (1 AVN excluded) Authors did not use “excellent” in outcomes
Dan Cosma [43]	6	6	0	0	2	NR	NR	3 metalware removal One re-slip after metalware removal requiring fixation	4 patients with unstable slips were excluded 8 had excellent and good results (stable and unstable slips) HHC
de Poorter [46]	61	78	2	NR	4	3	NR	5 THR	Long-term follow-up (18 years) HOOS (71) EQ5D score (0.83) EQ5D (VAS) 79%
Escott [31]	64	91	NR	NR	NR	NR	15 excellent 39 good 8 fair 2 poor		Long-term follow-up (20 years) HHS (84.9) SF12 (50) UCLA (7.3)
Gonzalez-Moran [47]	25	31	1	0	NR	NR	NR	1 wound infection 3 metalware problems	All received two weeks of skin longitudinal traction then pinning in situ without manipulation 22 cases had a single screw and 9 had 2 screws 11 acute, 6 acute on chronic and 14 chronic 1 preslip, 17 mild, 11 moderate and 2 severe
Guzzanti [23]	10	6	0	0	NR	NR	6 excellent		4 unstable slips were excluded from the analysis. 3 mild, 3 moderate and 0 severe Authors used the modified AO cannulated screw (HIT-MEDICA, Rimini, Italy) had a distal segment with the original six threads reduced to three which were 9 mm long and 6.4 mm in diameter
Holmdahl [55]	13	13	0	NR	NR	NR	NR		3 unstable slips were excluded. Authors used Hansson pin
Herman [48]	11	11	0	1	NR	NR	11 excellent 0 good 0 fair 0 poor	No further slipping	4 acute, 11 acute on chronic, and 6 chronic HHS (95 points)
Kenny [32]	40	53	0	1	NR	1	(31) 58% excellent (19) 36% good (2) 4% fair (1) 2% poor	1 sub-trochanteric fracture No further slipping	3 acute, 8 acute on chronic and 35 chronic 80% mild, 12% moderate and 2% severe HHC
Koval [49]	49	67	2	7	NR	2	NR	1 growing off fixation 1 stress fracture of the femoral neck	12 acute, 1 acute on chronic, 67 chronic 55 mild, 19 moderate and 6 severe 3 CRIF (1AVN)
Lim [50]	13	13	1	0			8 excellent 2 good 2 fair 0 poor 1 failure		All underwent preoperative traction All acute or acute on chronic Severity: mean 30° (range 0°–60°) Aadalen criteria
Novais [51]	15	15	1				3 excellent 1 good 1 fair 3 poor 7 failure	2 metalware problems 1 further slipping	All patients had stable severe slip revealed better deformity correction with the modified Dunn procedure compared with in situ pinning HHC
Souder [52]	NR	64	0	0	NR	NR	NR	3 metalware problems 1 infection 1 further slipping	Ganz surgical dislocation 7 unstable cause 3 AVN excluded
Ward [53]	42	53	0	0	NR	NR	NR	1 HO 2 metalware problems	2 acute, 3 acute on chronic and 48 chronic 19 mild, 25 moderate and 9 severe 5 CRIF
Wensaas [54]	14	16	0	0	NR	NR	NR	No metalware problem reported	2 unstable slips were excluded Authors used a modified Olmeda screw (De Puy)
Total (%)	NA	714	10	12	36		119 excellent 86 good 19 fair 10 poor 11 failure		AVN rate 1.4% (10/714) CL rate 2.0% (12/590) FAI 28.9% (36/121) OA 3.1% (6/195)

*AVN* femoral head osteonecrosis, *CL* chondrolysis, *CRIF* closed reduction and internal fixation, *NR* not reported or suboptimum reporting to provide useful information, *HHS* Harris hip score or modified Harris hip score; excellent 90–100 points; good 80–89 points; fair 70–79 points; and poor <70 points, *HHC* Heyman and Herndon classification, *WOMAC* Western Ontario and McMaster Universities Osteoarthritis Index, *FAI* femora-acetabular impingement, *OA* osteoarthritis

^a^ Satisfactory patients result based on closely related ratings such as Heyman and Herndon classification, Harris hip score or Iowa hip scores

**Table 7:** Studies of using multiple fine pins in stable slips

Study	Patients	Hips	AVN	CL	FAI	OA	Patient satisfaction^a^	Other complications	Notes
Aronson [44]	39	54	2	3	NR	18	27 excellent 13 good 0 fair 13 poor 1 failure	13 patients had pin protruding through the back of the neck	4 acute and 50 chronic 34 mild, 14 moderate and 6 severe HHC
Blanco [45]	NR	25	1	0	NR	NR	NR	8 metalware problems 1 growing off 4 reoperation	1 preslip, 4 acute, 6 acute on chronic, 12 chronic 11 mild, 9 moderate, 4 severe 7 CRIF
Carney [47]	NR	37	3	1	NR	NR			3 acute and 34 chronic Reduction and pinning resulted in a mean ISH of 75 points, 2 AVN, 1 CL. Pinning in situ resulted in a mean IHS of 85 points, 1 AVN, 0 CL IHS for chronic slips 86 and 93 for acute slips
Dreghorn [56]	NR	66	0	2	NR	0	NR	1 growing off fixation	51 mild, 14 moderate and 1 severe
Gonzalez-Moran [43]	28	31	0	3	NR	NR	NR	4 wound infection 9 metalware problems	1 acute, 4 acute on chronic and 26 chronic 0 preslip, 15 mild, 12 moderate and 4 severe
Zahrawi [42]	NR	60	0	2	NR	NR	49 excellent 6 good 0 fair 3 poor 2 failure	2 metalware problems 1 further slipping 3 wound infection 2 needed further surgery	Severity (mean slip angle 22) Chronicity and stability NR LOS 17 Duration of surgery 90 min Blood loss 250 ml HHC
Total	NR	273	6	11			76 (67%) excellent 19 (17%) good 0 (0%) fair 16 (14%) poor 3 (3%) failure		AVN rate 2.2% (6/273) CL rate 4% (11/273) FAI (NR) OA 15% (18/120)

*AVN* femoral head osteonecrosis, *CL* chondrolysis, *CRIF* closed reduction and internal fixation, *NR* not reported or suboptimum reporting to provide useful information, *HHC* Heyman and Herndon classification, *IHS* Iowa hip-rating system; excellent 90–100 points; good 80–89 points; fair 70–79 points; and poor ‹70points, *LOS* length of stay, *FAI* femora-acetabular impingement, *OA* osteoarthritis

^a^ Satisfactory patients result based on closely related ratings such as Heyman and Herndon classification, Harris hip score or Iowa hip scores

**Table 8:** Studies of physeal osteotomy in stable slip

Study	Patients	Hips	AVN	CL	FAI	OA	Patient satisfaction^a^	Other complications	Notes
Abu Amara [65]	NR	44	4	1	0	NR			See also PIS 92 unstable slips were excluded WOMAC (3.8) HHS (92.5)
Alshryda [3]	7	7	2	1	NR	NR	NR	Hip dislocation	15 unstable hips were excluded (5 AVN)
Barros [33]	23	23	3	2	NR	NR	9 excellent 9 good 1 fair 4 poor	1 metalware problem 0 infection	0 acute, 3 acute on chronic, 20 chronic 0 mild, 0 moderate, 23 severe MSC
Broughton [57]	115	115	14	14	1	17	67 good 9 fair 19 poor		0 acute, 38 acute on chronic, 77 chronic 0 mild, 15 moderate, 100 severe Patients satisfaction (G/F/B) in the acute on chronic (27/5/6); in the chronic with open growth plate (59/3/8) in the chronic slip with closed growth plate (1/1/5)
Carlioz [30]	26	27	0	3	NR	NR	20 good 3 fair 4 bad 3 failure	Septic arthritis	
Carney [35]	NR	14	3	6	NR	NR	NR		26 moderate or severe slips IHS for chronic slips 76 and 50 for acute slips
DeRosa [70]	23	27	4	8	NR	NR	0 excellent 19 good 4 fair 4 poor	2 loss of fixation	1 CRIF before PO went into AVN 0 mild, 0 moderate, 27 severe MSC
Dreghorn [56]	NR	3	1	0	NR	0	NR	1 wound infection	0 mild, 5 moderate and 6 severe
Diab [58]	11	11	2	0	1	NR	NR		
Dunn [59]	69	73	9	3	NR	2	55 good 6 fair 12 poor		Several hips were manipulated under GA somewhere else (CRIF) 0 acute, 33 acute on chronic, 40 chronic
Fish [60]	61	66	3	1	NR	6	55 excellent 6 good 2 fair 3 poor		0 acute, 16 acute on chronic, 50 chronic Chronic slips (0 mild, 23 moderate, 27 severe)
Fron [62]	46	50	6	3	NR	NR	34 excellent 10 good 2 fair 4 poor	2 hematomas 2 infections 3 pseudarthroses of the greater trochanter 1 HO	0 acute, 17 acute on chronic, 30 chronic 0 mild, 0 moderate, 50 severe
Jerre [63]	22	22	5	1	NR	6	5 excellent 4 good 1 fair 8 poor	4 THR 1 hip arthrodesis	1 acute, 1 acute on chronic, 20 chronic 10 mild, 6 moderate, 0 severe, 6 none HHC
Niane [64]	24	26	1	5	NR	NR	20 excellent and good 6 poor	The Postel Merle d’Aubigné (PMA)	Severity grade II and III only (grade I were excluded)
Nishiyama [65]	15	18	1	1			13 excellent 1 good 1 fair 0 poor		0 acute, 0 acute on chronic, 18 chronic 0 mild, 0 moderate, 18 severe
Szypryt [41]	23	23	4	0	NR	5	15 excellent 2 good 1 fair 4 poor	2 wound infection Metalware problems 10	1 acute, 16 acute on chronic, 6 chronic 0 mild, 0 moderate, 23 severe MSC
Velasco [66]	65	66	6	8			22 good 16 moderate (fair) 10 poor		8 acute, 29 acute on chronic, 29 chronic All moderate or severe (although Table 2 showed that angles <30° in five hips) Full set data in 48 hips
Total	NR	615	68	57			141 excellent 220 good 46 fair 78 poor 3 (6%) failure		AVN rate 10.4% (64/615) CL rate 9.2% (57/615) FAI rate 1.2% (2/170) OA rate 12.2 (36/294)

*AVN* femoral head osteonecrosis, *CL* chondrolysis, *CRIF* closed reduction and internal fixation, *NR* not reported or suboptimum reporting to provide useful information, *HHS* Harris hip score or modified Harris hip score; excellent 90–100 points; good 80–89 points; fair 70–79 points; and poor <70 points. *HHC* Heyman and Herndon classification, *IHS* Iowa hip-rating system; excellent 90–100 points; good 80–89 points; fair 70–79 points; and poor <70 points. *MSC* modified southwick criteria, *WOMAC* Western Ontario and McMaster Universities Osteoarthritis Index, *FAI* femora-acetabular impingement, *OA* osteoarthritis, *THR* total hip replacement

^a^ Satisfactory patients result based on closely related ratings such as Heyman and Herndon classification, Harris hip score or Iowa hip scores

**Table 9:** Studies of Ganz surgical dislocation in stable slips

Study	Patients	Hips	AVN	CL	FAI	OA	Patient satisfaction^a^	Other complications	Notes
Bali [67]	8	8	0	0	NR	NR	NR	2 non-unions requiring valgus intertrochanteric osteotomies	HHS: 92.5
Dan Cosma [43]	6	6	0	0	1	NR	6 excellent and good		One unstable slip excluded 10 pinned in situ HHC
Madan [68]	11	11	0	1	NR	NR	NR		17 unstable hips were excluded (4 AVN) 0 acute, 0 acute-on-chronic, 11 chronic 3 had previous operations HHS (90.3) NAHS(91.0)
Masse [69]	18	18	0	0		0	18 excellent 0 good 0 fair 0 poor 0 failure	1 metalware problem	2 unstable hips excluded (no AVN) 2 mild, 4 moderate, 12 severe HHS (98.2)
Novais [51]	15	15	1	NR	NR	NR	7 excellent 2 good 0 fair 5 poor 1 failure	2 metalware problems	HHC
Souder [52]	NR	10	2	1	NR	NR	NR	1 metalware problem	From a total of 17 hips, 7 were unstable, 2 of these unstable hips went into AVN
Ziebarth [70]	27	27	0	0	1		27 excellent 0 good 0 fair 0 poor 0 failure		25 patients from series A and 2 from series B 5 unstable/uncertain hips excluded 0 mild, 15 moderate, 12 severe HHS (96.5)
Total	NR	95	3	2			52 (87%) excellent 2 (3%) good 0 (0%) fair 5 (8%) poor 1 (2%) failure		AVN rate 3.1% (3/95) CL rate 2.5% (2/80) FAI rate 6.1% (2/33) OA rate 0% (0/18)

*AVN* femoral head osteonecrosis, *CL* chondrolysis, *NR* not reported or suboptimum reporting to provide useful information, *HHS* Harris hip score or modified Harris hip score; excellent 90–100 points; good 80–89 points; fair 70–79 points; and poor <70 points. *HHC* Heyman and Herndon classification, *NAHS* non arthritic hip scores, *FAI* femora-acetabular impingement, *OA* osteoarthritis

^a^ Satisfactory patients result based on closely related ratings such as Heyman and Herndon classification, Harris hip score or Iowa hip scores

## Outcomes

### Femoral head osteonecrosis

Data on the development of AVN was provided for 2162 hips. Of these, 109 hips (5%) developed AVN. The lowest rate of AVN was observed in the pinning in situ group using a single screw (1.5%) and the highest rate was observed in patients who underwent physeal osteotomy (11.1%). The different rates between interventions were statistically different [*χ* ^2^ test (*df* = 5): *P* < 0.001].

### Chondrolysis

An overall 108 out of 2071 hips (5.2%) developed chondrolysis (CL). The lowest rate of CL (1.3%) was observed in patients who underwent bone peg epiphysiodesis and the highest (20.5%) in patients who were treated with hip spica. The different rates between interventions were statistically different [*χ* ^2^ test (*df* = 5): *P* < 0.001]. Table 4 shows a pooled summary of the AVN rates among various interventions.

### Femoro-acetabular impingement

Six studies (324 hips) provided useful data on the rate of FAI [43, 46, 57, 58, 70, 71]. These studies investigated pinning in situ, physeal osteotomy and Ganz surgical dislocation. The FAI rates were 29.8, 1.5 and 6%, respectively. The difference was statistically significant [Fisher exact test (*df* = 2): *P* < 0.001]. One study [71] reported the presence of radiological signs of FAI in 30 of 37 hips that were treated with pinning in situ. The study was not explicit about their impingement symptoms. Sensitivity analysis by excluding the study showed that the FAI rate was 7, 1.5 and 6%, respectively; a non-significant difference [Fisher exact test (*df* = 2): *P* = 0.13].

### Osteoarthritis

The overall OA rate was 11% with the lowest rate seen in patients who underwent Ganz surgical dislocation (0%), followed by PIS (3.1%). Hip spica was associated with the highest OA rate (52%). The variation in the OA rates among various interventions difference was statistically significant [Fisher exact test (*df* = 5): *P* < 0.001].

### Patient satisfaction rates

**Fig. 2:**
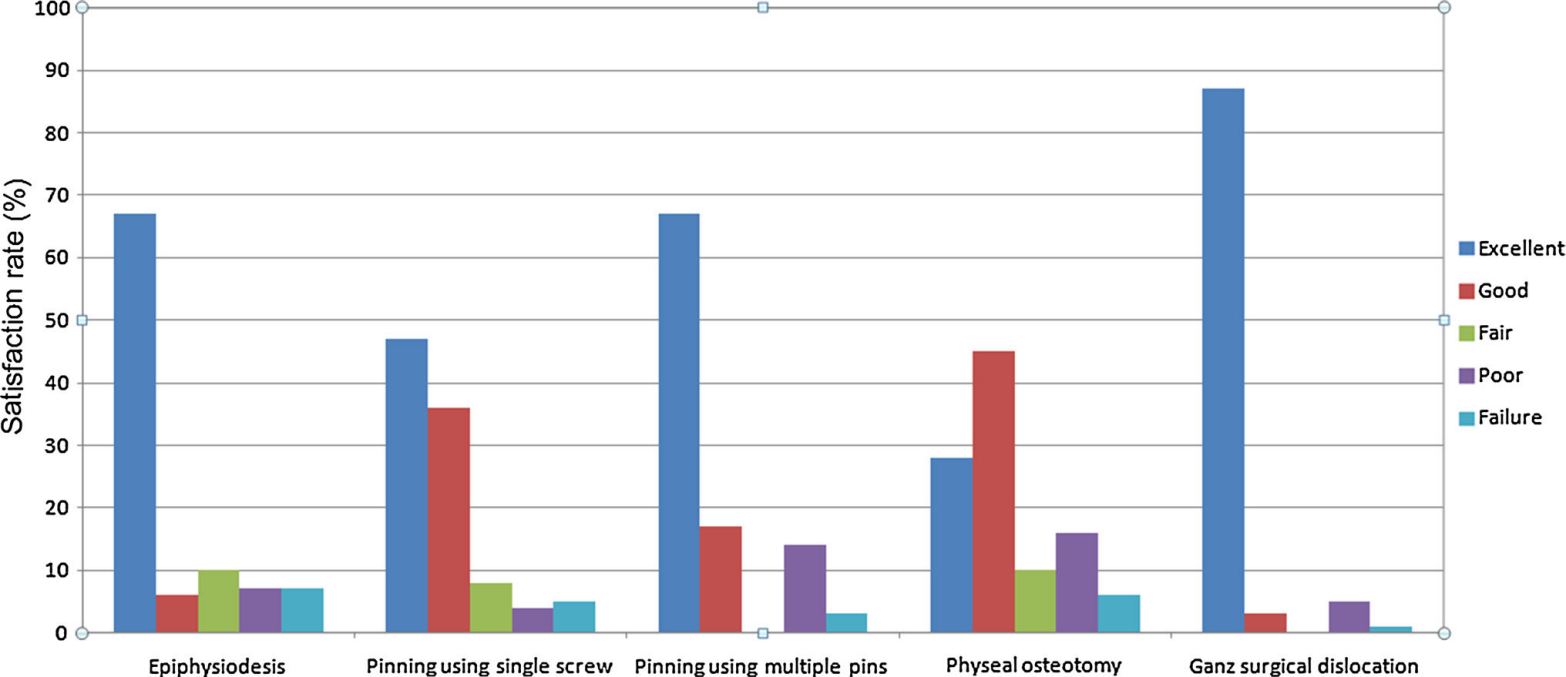
Satisfaction rates among various interventions to treat slipped capital femoral epiphysis

Patient satisfaction scores were reported for all interventions apart from hip spica. Most included studies used closely related scores which were categorised into an ordinal scale of (excellent, good, fair, poor and failure). These are summarised in Fig. 2. Visual analysis of the graphs favours Ganz surgical dislocation.

### Continued growth

Three studies reported on screws that allow for continued growth. Guzzanti [23] reported on 12 patients (6 with stable SCFE) who were treated using a modified AO cannulated screw (HIT-MEDICA, Rimini, Italy). The distal segment has 3 rather than the original six threads. Holmdahl [55] reported on 13 patients (10 with stable SCFE) who were treated with Hansson hook pins. The Hansson hook pin is a smooth 6.5-mm-diameter pin with a central hook that offers secure anchorage in the epiphysis and the smooth pin allows the femoral neck to continue to grow. Wensaas [54] reported on 14 patients (16 stable SCFE) who were treated with a modified Olmeda screw (De Puy). The screw has a shorter thread segment. The three studies used various measures to assess growth and remodelling. They showed that growth and remodelling continued when these screws were used.

## Discussion

Several aspects of slipped capital femoral epiphysis remain controversial. Loder’s work has been instrumental in changing our understanding and approach to the management of the condition when he introduced the concept of “slip instability”, which was fundamental in better understanding certain aspects of the condition. Two different types of SCFE became apparent; unstable slips where the patient cannot ambulate even with crutches, and stable slips where the patient can ambulate. Loder showed that AVN developed in 47% of unstable slips but none of the stable hips. This finding has been replicated by others [3–6, 71].

These two types of SCFE behave differently in terms of presentation, progress and complications; hence treatments are likely to be different. For this reason, we approached them as two different conditions to highlight these differences [26]. In our previously published review, open reduction and internal fixation using the Parsch technique [26, 72] stood out as the best current technique to treat unstable slipped capital femoral epiphysis. In this review, five outcomes were analysed to compare various interventions: AVN, CL, FAI, OA and patient satisfaction rates. Pinning in situ and Ganz surgical dislocation are shown to be superior to other interventions in treating stable slips (Table 3).

The review confirmed that the rate of AVN in stable slips is much lower than that in unstable slips (5.1 versus 21%) [26]. The type of surgical intervention is an important risk factor. Pinning in situ was associated with the lowest AVN rate (1.4%). Moreover, the CL, FAI and OA rates were relatively low in patients who underwent PIS. These were not translated into high patient satisfaction rates among these patients, with only 47% reporting an “excellent” outcome. In contrast, 87% of patients who underwent Ganz surgical dislocation reported an “excellent” outcome. The Ganz surgical dislocation was associated with an AVN rate of 3.3%; double that observed in pinning in situ. Of note, 5 of the 7 studies which investigated the Ganz dislocation reported a AVN rate of 0%. The overall mean AVN rate for this technique is hence derived from the two remaining studies and therefore the actual AVN rate for this procedure may in reality be lower than 3.3%. As a relatively new procedure and with small patient numbers, there may also be a substantial learning curve associated with this technique.

When non-threaded pins and wires were used, the neck commonly continues to grow and this would be a great advantage. However, stabilisation using multiple pins was not found to provide advantages over pinning using a single screw, with substantially higher AVN, CL, FAI and OA rates. Moreover, with continued growth there is a risk that the anchorage in the epiphysis will be lost and repeat fixation will be required. Further growth of the femoral neck is less likely to occur if a screw is inserted in compression mode with the head abutting the lateral femoral cortex, causing physiodesis [73]. Three studies [23, 54, 55] showed that screws with special design allowed growth to continue; however, these were small studies (37 patients) with no comparator. The literature search identified 6 other studies that used screws which allow continued growth and reported a favourable outcome on neck growth; however, these studies could not be included in our review because we were not certain about the stability of the slips.

Physeal osteotomy using Fish or Dunn techniques was associated with high AVN, CL, FAI and OA rates and only 28% reported an excellent outcome. Bone peg epiphysiodesis has not been favoured by the orthopaedic community because of the associated blood loss, donor site morbidity, length of surgical time and length of stay. Although the AVN and CL rates were relatively low, patients’ satisfaction rates were not impressive with only 67% reporting an excellent outcome and 14% reporting a poor outcome or failure. Although the reported OA rate was high (23.3%), this was derived from a single study which could be an outlier.

Hip spica was found to be the worst treatment, with poor AVN (9%), CL (20.5%) and OA (53%) rates. This probably explains why this treatment modality has largely been abandoned in the management of SCFE.

Timing of surgery and severity of the slip are two factors that we intended to study; however, included studies did not provide useful data to inform the effect of these factors on the selected outcomes. It is our observation that timing of surgery is not as critical as in unstable slips but the severity of slips plays a role in final outcomes and patient satisfaction rates. However, this remains to be proven.

The review was conducted with the intention of doing a trial-based meta-analysis. Disappointingly, only case series and controlled studies were found and form the basis of this review. Some studies were published more than once with or without extra information. Thorough considerations have been taken when including data from such studies. Authors were often contacted for further clarification and data provision. Despite our best effort to produce a high quality review, the qualities of the included studies remain the major weakness of this review. Yet, this is the largest systematic review that has addressed this hot topic and which explains the current trends in treating slipped capital femoral epiphysis.

Another inherent bias that must be considered when reading our findings is the fact that pinning in situ was used across the whole severity spectrum of SCFE and this was not the case with Ganz surgical dislocation. Ganz surgical dislocation is more invasive than PIS and has been reserved for patients with severe stable slips. In such cases, the merits and risks of this technique should be discussed with the parents: an excellent reported patient satisfaction but a higher incidence of AVN, respectively.

In summary, the review supported our views that stable and unstable slips behave differently and require different treatments. For an unstable SCFE, open reduction and internal fixation on an urgent basis (within 24 h) is shown to be associated with the best outcome [26]. For a stable SCFE, pinning in situ is recommended for mild and, to a lesser extent, moderate slips. Screws that allow continued growth may be superior to standard screws. Ganz surgical dislocation is recommended for severe slips provided patients and parents agree to take the higher AVN risk for better satisfaction and the surgical expertise is available.
